# Plant terrestrialization: an environmental pull on the evolution of multi-sourced streptophyte phenolics

**DOI:** 10.1098/rstb.2023.0358

**Published:** 2024-09-30

**Authors:** Cäcilia F. Kunz, Sophie de Vries, Jan de Vries

**Affiliations:** ^1^ Department of Applied Bioinformatics, Institute for Microbiology and Genetics, University of Goettingen, Goettingen 37077, Germany; ^2^ Campus Institute Data Science (CIDAS), University of Goettingen, Goettingen 37077, Germany; ^3^ Goettingen Center for Molecular Biosciences (GZMB), Department of Applied Bioinformatics, University of Goettingen, Goettingen 37077, Germany

**Keywords:** terrestrialization, evolution, streptophyte algae, secondary/specialized metabolism, phenylpropanoids, lignin

## Abstract

Phenolic compounds of land plants are varied: they are chemodiverse, are sourced from different biosynthetic routes and fulfil a broad spectrum of functions that range from signalling phytohormones, to protective shields against stressors, to structural compounds. Their action defines the biology of land plants as we know it. Often, their roles are tied to environmental responses that, however, impacted already the algal progenitors of land plants, streptophyte algae. Indeed, many streptophyte algae successfully dwell in terrestrial habitats and have homologues for enzymatic routes for the production of important phenolic compounds, such as the phenylpropanoid pathway. Here, we synthesize what is known about the production of specialized phenolic compounds across hundreds of millions of years of streptophyte evolution. We propose an evolutionary scenario in which selective pressures borne out of environmental cues shaped the chemodiversity of phenolics in streptophytes.

This article is part of the theme issue ‘The evolution of plant metabolism’.

## 1. Introduction

For success on land, plants must be able to cope with various abiotic and biotic challenges. Although numerous lineages of algae have conquered land, none, except for the monophyletic clade of Embryophyta, have radiated in diverse habitats and risen above their substrate. Roughly 590 million years ago, embryophytes emerged from the streptophyte algae, which occur mainly in freshwater and terrestrial habitats [1–4]. Concepts that aim at explaining the unique evolutionary origin of land plants pose that the algal progenitor of embryophytes had a fortuitous combination of biochemical, morphological and physiological characteristics that aided in ushering in the conquest of the terrestrial habitats [5]. These emerged as exaptations or adaptations [6] of either a terrestrial algal conqueror [7] or lingering genetic potential [1,6,8]. Indeed, a plausible scenario is that such an ancestor dwelled in freshwater environments with frequent wet–dry cycles, imposing a selective pressure on adaptations necessary for terrestrialization. What is more, freshwater environments provide a gentler ecological gradient compared with marine environments [5]. On land, additional factors contributed to embryophytic success, such as microbial communities that probably aided the earliest land plants in conquering this novel environment by forming symbiotic relationships [9,10].

The clarification of the exact phylogenetic relationship between land plants and streptophyte algae has motivated a series of comprehensive phylogenomic work over the last decade [11–14]. These analyses underpinned the deep split between the Chlorophyta, the recently recognized Prasinodermatophyta [15], and the Streptophyta. The latter contains the monophyletic Embryophyta and paraphyletic streptophyte algae, which can be grouped into two grades, KCM and ZCC [16]. The ZCC grade contains the classes Zygnematophyceae, Coleochaetophyceae and Charophyceae; the KCM grade Klebsormidiophyceae, Chlorokybophyceae and Mesostigmatophyceae. ZCC and Embryophyta form a monophylum called Phragmoplastophyta. Thus, the last common ancestor (LCA) of land plants and algae was a phragmoplastophyte and all analyses agree that the closest algal relatives of land plants are the Zygnematophyceae [11–14]. On land, the earliest land plants—the stem embryophyte, the first common ancestor of all land plants—acquired multiple adaptations before the LCA of all extant embryophytes [1].

Previous genomic studies have highlighted metabolic inventions associated with diversification during the conquest of land [17]. Yet phylogenomic and first metabolomic insights highlight that major groups of land plant metabolites (e.g. precursors of jasmonates, auxin, ABA, phenolics, including flavonoids, salicylic acid and other benzoates) are present in streptophyte algae and thus were likely already present in a common ancestor shared between land plants and algae [18–26]. Here, we review recent advances in our understanding of the evolution of specialized phenolic compounds and discuss how plant terrestrialization and the environmental stressors that the terrestrial habitat has in store contribute to phenolic diversity in algae and land plants.

## 2. Phenolic compounds and stress on land with a focus on UV-B

Phenolic compounds are key specialized metabolites of land plants and algae. They have diverse roles as pigments, antioxidants, signalling agents, structural elements (like lignin) and defence metabolites. Their accumulation (and often the expression of the enzymes that give rise to them) is induced by biotic and abiotic stresses, such as herbivores, pathogens, temperature fluctuations, pH changes, saline stress, heavy metals and UV radiation [27].

Stress resilience is key for the lasting conquest of land by streptophytes—both more than 500 million years ago as well as nowadays [1]. Terrestrial stressors include, among others, water scarcity, rapid shifts in temperature, high photosynthetically active irradiance (PAR) and ultraviolet radiation (UVR). In an aquatic habitat, the overlying water column protects the sublittoral algae against most of the radiation in aquatic ecosystems. Even though UVR penetrates pure water deeper compared with other radiation, freshwater usually teems with other organisms and dissolved compounds, such as phenolics, which filter and absorb the UVR efficiently [28]. For terrestrial microorganisms, the formation of biological soil crusts (BSCs) forms an added layer of protection against high irradiance [29]. Yet, the number of high-energy wavelengths of UV-B (280−315 nm) radiation was higher during the evolution of the first land plants than it is currently [30–33]. To what extent and which composition barriers to UV radiation such as BSCs were already present in the early colonization of land is another matter. Damaging UV-B radiation can directly induce alterations of biomolecules like DNA and proteins, directly impact the function of photosynthesis, and indirectly via metabolically toxic reactive oxygen species (ROS); thus, UV-B radiation was (and persists to be) a major challenge for photosynthetic organisms on land [34].

Driven by the requirement for light of phototrophs, protective mechanisms have evolved to ensure enough visible light for photosynthesis reaches the chloroplasts, while UVR damage is avoided [34]. These mechanisms include DNA repair processes, UVR avoidance, the biosynthesis of antioxidants and UV-screening compounds [35]. The latter may absorb the energy of UVR, acting as mere shields, disposing of the previously harmful energy in the form of heat, thereby preventing damage to essential biomolecules [34]. The absence of such mechanisms would result in harmful effects, such as reduced or inhibited growth [36,37], reproduction, survival [38], protein stability in photosystem II (PSII), pigmentation [39] and activity of the key enzyme for CO_2_ fixation, ribulose-1,5-bisphosphate carboxylase-oxygenase (RuBisCO) [40]. Therefore, organisms need those UV-shielding specialized metabolites to be produced when necessary.

**Figure 1.**
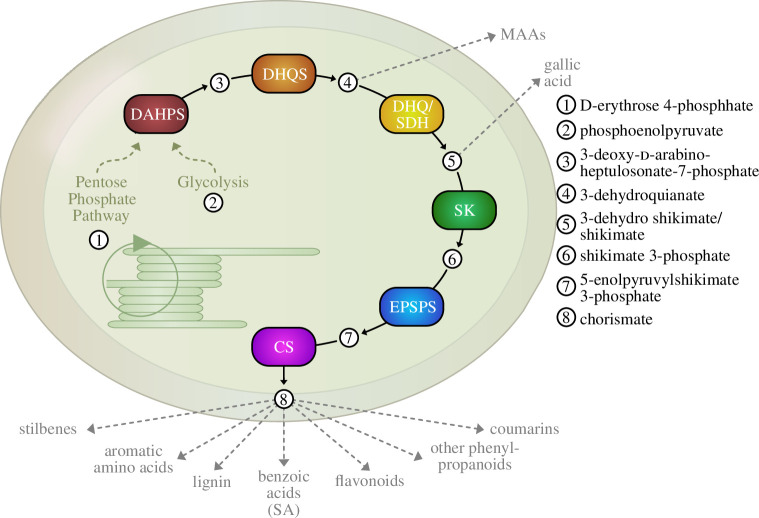
Shikimate pathway. Compounds of glycolysis and the pentose phosphate pathway funnel into the shikimate pathway in the chloroplast, linking primary to spezialized metabolism. The end product (chorismate) is the first compound for multiple pathways, leading to a plethora of compounds. DAHPS: 3-deoxy-d-arabino-heptulosonate-7-phosphate synthase; DHQS: 3-dehydroquinate synthase; DHQ/SDH: 3-dehydroquinate dehydratase/shikimate dehydrogenase; SK: shikimate kinase; EPSPS: 5-enolpyruvylshikimate 3-phosphate synthase; CS: chorismate synthase. Inspired by Tzin *et al*. [41].

There are several routes for stress-induced (as well as constitutive) production of phenolics in land plants and algae, but the foremost source for a plethora of compounds is the shikimate pathway (figure 1) [42]. Named after the toxic Japanese shikimi flower (*Illicium anisatum*), the shikimic acid serves as a link between primary and specialized metabolism in land plants. The pathway encompasses seven reaction steps, beginning with an aldol-type condensation of phosphoenolpyruvic acid (PEP) and d-erythrose-4-phosphate leading to chorismic acid as its final product [43]. Conversions of this product yield the aromatic amino acids l-phenylalanine, l-tyrosine and l-tryptophan, essential for protein biosynthesis. In plant metabolism, these amino acids are not only vital for protein synthesis but also act as precursors for diverse specialized metabolites crucial for plant growth and adaptation to their environment—and for their general structure [44]. This is because the shikimate pathway directly feeds into phenylpropanoid biosynthesis, yielding cinnamic acid as a central metabolite. Branches of the products of the phenylpropanoid pathway leading to, in turn, coumarins, flavonoids, benzenoids, stilbenes, suberin and precursor of lignin [45], exhibiting great structural and chemical versatility and contributing to the complex chemodiversity of land plants. Compounds derived from branches of this pathway, such as mycosporine-like amino acids (MAAs), flavonoids and coumarins, are induced upon UVR and act as sunscreens [46,47]. Flavonoids also include some ROS-scavenging molecules like anthocyanins, produced from *para*-coumaroyl-CoA, a phenylpropanoid pathway compound, in the flavonoid pathway [48].

## 3. Algae produce phenolics

Especially UVR is an abiotic factor that induces the biosynthesis of phenylpropanoids—and their flow into flavonoid biosynthesis [49]. This link appears conserved across land plants [46,48,50]. In Klebsormidiophyceae, some species display remarkable characteristics in their response pattern to abiotic stressors. While Klebsormidiophyceae have deeply radiated more than 800 million years ago [8], they share the production of MAAs such as Klebsormidin a and b (324 nm MAAs) [51,52], which serve a photoprotective function and are produced upon UVR [51]. They are the best-characterized MAAs in streptophyte algae. MAAs can derive from two pathways, the shikimate pathway and the pentose phosphate pathway [53]. Only MAAs derived from the shikimate pathway are required for photoprotection in cyanobacteria [54], whereas products of the pentose phosphate pathway may have other biological functions [53], such as in osmotic stress regulation and desiccation tolerance [55]. Thus, within this review, we focus on shikimate pathway-derived MAAs. In other streptophyte algal genera, like the Zygnematophyceae, MAAs are not known to be present [56]. Since MAAs are a major UV-protection strategy in chlorophyte algae [37] this suggests convergent evolution [1]. Zygnematophyceae use phenolic compounds as UV protection, such as colourful complexes of gallic acid derivatives with ferric iron, leading to purple pigment accumulation in the vacuoles of *Zygogonium ericetorum* [57,58]. Glycosylated purpurogallin derivatives lead to red-brown pigments in cryophilic *Ancylonema alaskanum* (formerly *Mesotaenium berggrenii* var. *alaskanum*) [59,60]. In addition to these observed intracellular accumulations of sunscreens, a species of the genus *Serritaenia* (Zygnematophyceae) forms UV-protective extracellular mucilage [61]. This is very unusual for algae, as the intracellular accumulation of sunscreens is more common [59,62].

In land plants, the production of phenolics is mediated by the UV-B-specific photoreceptor UV-resistance Locus8 (UVR8), which perceives UV-B and subsequently directs the response, such as initiating the biosynthesis of flavonoids [46,63–66]. Due to the conservation of this mechanism across Embryophyta, the UVR8-mediated flavonoid induction may have been present in the earliest land plants [46]. In *Arabidopsis*, the inactive dimer is constantly present and activated upon UV-B absorption [67,68]. The thereby cleaved active monomer accumulates in the nucleus and its binding to E3 ubiquitin ligase CONSTITUTIVE PHOTOMORPHOGENIC 1 (COP1) triggers response pathways [68,69]. The COP1 complex stabilizes the bZIP transcription factor ELONGATED HYPOCOTYL 5 (HY5), which is a transcriptional regulator of UV-B photomorphogenic response genes [70,71]. These genes orchestrate responses in the protection of photosynthetic machinery, DNA repair, as well as the production of UV-B-absorbing phenylpropanoids and flavonoids [46,72,73]. In the liverwort *Marchantia polymorpha*, UVR8 was found to be produced 12 h after exposure to UV-B radiation [46]. UVR8 has been shown to be present in Zygnematopyhceae [74]; the regulation of phenolic production upon UV-B irradiation in streptophyte algae remains a characteristic to be further investigated to elucidate the potential regulatory repertoire of the LCA of Embryophytes and streptophyte algae.

## 4. Evolution of a conserved chassis and the options for divergence

The universal usage of the phenylpropanoid pathway during stress response in embryophytes was thought to have emerged early during their evolution. Yet, homologues to most—but not all [22]—enzyme-coding genes of the core pathway were detected in many streptophyte algae species [19,20,75,76]. These patches notwithstanding, phenolic compounds that include diverse flavonoids (e.g. kaempferol-3-glucuronide, rutin and more)—and even lignin(-like) imbuements of the cell wall—have been detected in even those species where the genetic basis for their production is enigmatic [22,77–80]. While the enzyme families involved have radiated and diverged, especially during the evolution of embryophytes, a series of homologues of these enzyme families were pinpointed in streptophyte algae [19]. The high evolutionary versatility indicated by these radiations suggests functional divergence of the pivotal enzymes involved in this pathway [19]. While the functionality of many of these genes remains to be elucidated, their presence depicts the ancestral gene pool from which the embryophytic stress response genes emerged [1,6]. Versatility in the evolution of promiscuous enzyme families can be considered a key component of the evolutionary substrate from which functions with a selective advantage might have been recruited during the earliest steps of plants on land [1,76].

As mentioned, chemodiversity in plant phenolics foremost starts with the shikimate pathway (figure 1). The shikimate pathway consists of seven conserved enzymatic steps that are well-characterized [81] and will thus here be only briefly mentioned; they include 3-deoxy-d-arabino-heptulosonate-7-phosphate synthase (DAHPS), 3-dehydroquinate synthase (DHQS), plant-specific bifunctional enzyme complex [41] 3-dehydroquinate dehydratase/shikimate dehydrogenase (DHQ/SDH), shikimate kinase (SK), 5-enolpyruvylshikimate 3-phosphate synthase (EPSPS), and chorismate synthase (CS). The enzymes involved in the shikimate pathway likely derive from three different prokaryotic sources [82,83]. DHS likely has an α-proteobacterial origin with subsequent endosymbiotic gene transfer (EGT), SK and CS are of cyanobacterial origin via EGT [83,84], whereas DHQS, DHQ-SDH and EPSPS might be of other prokaryotic origin, with a complex evolutionary history that could involve horizontal gene transfer (HGT), either directly to the base of Archaeplastida or first a transfer to the cyanobacterial progenitor and subsequent acquisition via EGT [82,84], with the phylogenetic signals having been muddled due to inherited chimerism [85]. At the base of seed plants, a gene duplication event of SDH followed by a few mutations led to the emergence of the neofunctionalized quinate dehydrogenase (QDH) gene family. Therefore, in angiosperms and gymnosperms, the product of DHQS can be funnelled either into the shikimate pathway via DHQ/SDH or into the side branch of quinate metabolism via QDH. The resulting quinate can either be reversibly reconverted to dehydroquinate to fuel the shikimate pathway or lead to complex specialized metabolites [86]. The first reaction in the shikimate pathway is the defining committing step: the condensation of phosphorylated compounds, phosphoenolpyruvic acid (PEP) from the glycolytic pathway, and d-erythrose-4-phosphate from the pentose phosphate cycle, forming 3-deoxy-d-arabino-heptulosonic acid 7-phosphate (DAHP). DAHPS catalyses the transfer of the enolpyruvyl moiety from PEP to d-erythrose 4-phosphate [87,88] and thus represents the link between primary metabolism with specialized metabolism. Subsequent reactions lead to the production of chorismic acid. While earlier branching points exist, e.g. those leading to gallic acid [89], most of the diverse phenolic compounds arise from chorismic acid. From this, several important pathways commence, some of which are highlighted in the following. But it should be noted that both these early branching points (e.g. the mentioned one leading to gallic acid), as well as early products from the pathway (chlorogenic acid), have relevance in stress response [90,91].

### (a) Salicylic acid pathway

There are two major routes for SA biosynthesis that also traverse other important compounds. The first encompasses the route from isochorismate to SA requiring isochorismate synthase (ICS) and the Gretchen-Hagen enzyme AVRPPHB SUSCEPTIBLE 3 (PBS3), which catalyses the fusion of glutamate to isochorismate. This is followed by either a spontaneous decay or active conversion into SA, the latter of which is catalysed by ENHANCED PSEUDOMONAS SUSCEPTIBILITY 1 (EPS1) in *Arabidopsis thaliana* [92,93]. EPS1 originated from an Arabidopsis-specific duplication, yet closely related co-orthologues in other Brassicaceae exist, while other angiosperms have only more distantly related homologues [19,93]. Thus other angiosperms may rely fully on a non-enzymatic spontaneous decay to synthesize SA. The second route, also employed in several angiosperms [94–98], involves the initial steps of the phenylpropanoid pathway to convert phenylalanine to cinnamic acid. From there, it is funnelled into the synthesis of benzoic acid which can be converted into hydroxybenzoic acids, including SA [99]. This can span across compartments: cytosol, mitochondria and peroxisome—or a combination thereof [99]. SA can be detected across the green lineage [18,26], yet which of the two routes is predominantly used across the green lineage is only scarcely investigated. Jia and colleagues [26] demonstrated that lack of ABNORMAL INFLORESCENCE MERISTEM1 (AIM1), an enzyme employed in the route towards benzoic acid, resulted in reduced levels of SA in *Chlamydomonas rheinhardtii,* while the *ICS* knockdown did not show a significant reduction. Moreover, MenC and/or MenD fusions to ICS are observed in representatives of bryophytes and lycophytes as well as chlorophyte and streptophyte algae [100]. MenC and MenD are domains of PHYLLO, a protein encoded by a gene that was derived from a gene fusion of the likely EGT-derived Men operon [101]. ICS derives from a duplicated MenF domain [101]; thus, one can envision a duplication of *PHYLLO* that was during evolution reduced to give rise to an ICS-encoding gene. As such isochorismate of algae and several land plants may be funnelled into phylloquinone biosynthesis instead of SA. In these organisms, SA could mainly derive from benzoic acid. Among land plants, the water fern *A. filiculoides* is the only one that has lost *ICS,* yet SA is produced [100]. This highlights the variability of land plants in the production of SA. Overall, the current data suggest that in chlorophytes benzoic acid was the major source of SA. When ICS became employed is currently not clear. Yet, a complete reliance on the ICS pathway has been demonstrated only for *Arabidopsis* [102]. Whether this is a Brassicaceae-specific trait while other land plants rely on redundancy remains to be investigated. Here, diversity in land plants as well as streptophyte algae needs to be assessed to determine the capabilities of SA synthesis in LCA of embryophytes.

Both routes, however, require chorismate, which is then either converted into isochorismate or phenylalanine. The latter step has both a cytosolic as well as a plastidial route, employing either the phenylpyruvate or arogenate pathway. In the plastid, chorismate mutase 1 (CM1) converts chorismate to prephenate and then to arogenate via a prephenate aminotransferase [103]. Arogenate dehydratase (ADT) converts arogenate to phenylalanine [104]. In the cytosol, CM2 converts chorismate to prephenate, which is then converted into phenylpyruvate by a prephenate dehydratase (PDT) [105]. Cytosolic PhPDT in *Petunia hybrida* is produced from an alternative transcription start site within the gene encoding the plastid localized PhADT3 [105]. Phenylpyruvate is the substrate for phenylpyruvate aminotransferase, which catalyses the reaction to phenylalanine [106]. Wounding induces alternate transcription of the PhADT3 encoding gene, which then shifts its location from the chloroplast to the cytosol [105]. With this switch, phenylalanine is now predominantly produced in the cytosol rather than the chloroplast. The exact biological relevance of the shift of the phenylalanine pool location is not yet clear. However, this means that the metabolic context of cytosol and chloroplast is changed with respect to a key metabolite that sits at the intersection of many wounding and defence-associated pathways. This could reduce regulatory constraints, as cytoplasmic phenylalanine can directly be funnelled into the wound- and defence-associated response pathways. This may also affect the levels of SA in plants that, during biotic interactions, rely on phenylalanine-dependent pathways for SA production. Indeed, the plastidial synthesis of isochorismate versus a shifted synthesis of phenylalanine to the cytosol may even trigger a shift in the SA source.

There is an interesting pattern to be seen here. Namely, that upon biotic-associated stress responses (e.g. wounding and direct pathogen challenge), some plants shift the location and/or pathway to produce key metabolites for defence response. In *P. hybrida*, wounding shifts phenylalanine synthesis to the cytosol, changing the main contribution to the phenylalanine pool from plastidial to cytosolic. Soybean partially involves benzoic acid-derived SA in its defence responses, instead of fully relying on isochorismate as the source. This begs the question of whether not only the compound produced but also the way it is produced plays a role in how defence responses bear out. That is, the entire metabolic context matters, in which defence metabolites occur. It can be conceived that environmental cues trigger a specific makeup of the metabolic network by shifting metabolic availability within the network and the cellular space.

### (b) Core phenylpropanoid pathway

**Figure 2.**
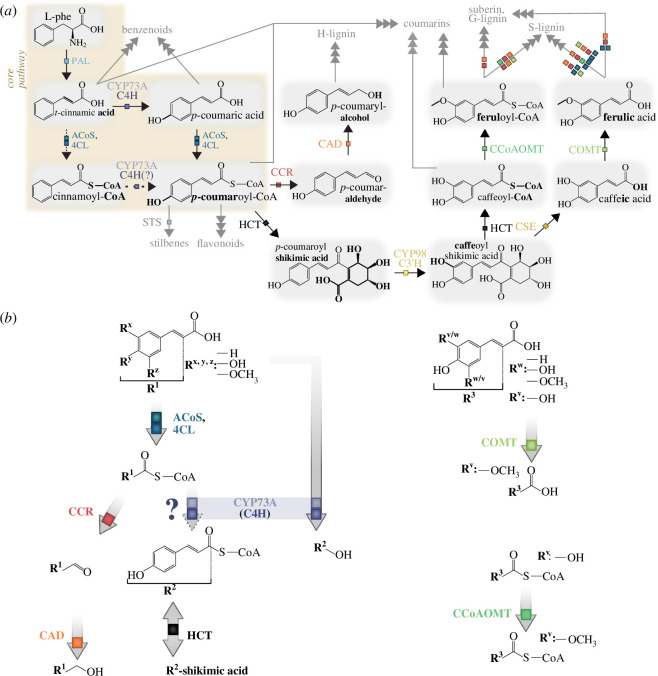
Schematic views of selected reactions within the phenylpropanoid pathway representing the modular activity of the enzyme family. (*a*) Core phenylpropanoid enzyme reactions are indicated by a brown background. Enzyme families are colour-coded based on identity and represented by a coloured square. Changes occurring due to enzyme activity are highlighted in bold, in the direction of arrows. Dotted lines indicate putative/ambiguous steps in the pathway, except for ACoS, 4CL, where it indicates that cinnamate is a poor substrate for plant 4CLs. Branches to other pathways are indicated by grey arrows and writing. (*b*) Enzyme families (grey arrows with colourful boxes) convert compounds in a modular manner, dependent on certain characteristic functional groups. Note that for CYP73 (C4H) *R*_x, y, z_ are -H. In the case of COMT and CCoAOMT, at least one of the rest groups must be an -OH (R_v_) on which the enzyme acts, whereas the other can be either -H, -OH or -OCH_3_ (*R*_w_). PAL: phenylalanine ammonia lyase; ACoS: acyl-CoA synthetases; 4CL: 4-coumarate-CoA ligase; CCR: cinnamoyl-CoA reductase; CAD: cinnamyl alcohol dehydrogenase; CYP73A: cytochrome P73A; C4H: cinnamate 4-hydroxylase; HCT: hydroxycinnamoyl-CoA shikimate/quinate hydroxycinnamoyltransferase; CYP98: cytochrome P98; C3’H: *p*-coumaroyl ester/quinate/shikimate 3′ hydroxylase (CYP98); CSE: caffeoyl-5-*O*-shikimate esterase; COMT: caffeate *O*-methyltransferase; CCoAOMT: caffeoyl-CoA *O*-methyltransferase; STS: stilbene synthase.

The plant phenylpropanoid pathway commences with the conversion of phenylalanine and/or tyrosine into cinnamate and/or *p*-coumarate, catalysed by phenylalanine ammonia lyase (PAL) and/or the bifunctional phenylalanine tyrosine ammonia lyase (PTAL) enzymes (figure 2*a*) [107]. Initially believed exclusive to land plants, recent discoveries of PAL-like genes in streptophyte algae, such as *Klebsormidium nitens*, challenge this notion [8,19,75]. Streptophyte PAL homologues fall into two distinct clades, one of land plants and one of Klebsormidiales and bacteria [8,19]; additionally, ammonia lyase homologues of the streptophyte alga *Chara braunii* cluster with histidine ammonia lyases (HALs) [19]. Very recently, characterization of one of these homologues in *Chara braunii* showed that—despite this phylogenetic affinity to HALs—the gene product has bona fide PAL activity [108]. A clade of fungal PALs clustering with land plant PALs speaks of a complex evolutionary history. However, the distinct clade of streptophyte PALs and the lack of PAL homologues in any other streptophyte algal lineage, except for Klebsormidiales, would suggest a distinct acquisition of PAL (or alternatively frequent loss with only two streptophyte lineages retaining PAL homologues). Independent of which is more likely, phenylpropanoid-derived compounds are produced by streptophyte algae (e.g. [22]) and thus some feed-in into a phenylpropanoid pathway must exist. The recent data by Schwarze & Petersen [108] point to some flexibility in ammonia lyases and open up the question of whether during streptophyte evolution several routes into the phenylpropanoid pathway have independently emerged.

Following the initial committed step into the phenylpropanoid pathway by PAL, the synthesis of cinnamate, two pathways emerge, the major one involving cinnamate conversion into *p*-coumarate through cinnamate 4-hydroxylase (C4H)—which occurs across vascular and non-vascular land plants (figure 2*a*) [109–111]. C4H is a member of the extremely diverse cytochrome P450 (CYP450) enzyme family found across all domains of life. In land plants, CYP450s, specifically the CYP73A subfamily including C4H, have undergone significant duplication and subfunctionalization, contributing to embryophyte metabolic specialization. Notably, C4H orthologues are identified only in land plants and not in streptophyte algae [19]. However, UHPLC-MS/MS analysis has detected the C4H product, *p*-coumarate, in phylodiverse algae [112], indicating an alternative route independent of C4H. This could be through direct tyrosine transformation by an enzyme with PTAL activity (noting that neither PAL nor PTAL candidates have been found in streptophyte algal lineages, except the PAL-like sequences of the Klebsormidiales and the recently characterized PAL of *Chara braunii* [108]) or a distinct, highly divergent C4H homologue. As it stands, the majority of CYP450 sequences are not characterized—they are potential candidates for the algae CYP450 enzyme family catalysing the C4H-function, especially given the high promiscuity of the group (and resulting evolutionary versatility [113]). According to KEGG [114,115], C4H (EC1.14.14.91 (previously EC1.14.13.11)) is able to catalyse a second reaction, the conversion of cinnamoyl-CoA to *p*-coumaroyl-CoA (R08815). Yet, our literature research suggests that this needs further scrutiny. While the paper referred to on KEGG for this reaction [116] does not show its occurrence, several papers state this reaction could be catalysed by an enzyme related to C4H, namely cinnamoyl-CoA 4-hydroxylase (CC4H) (with the first mention that we found being the work of Rastogi *et al*. [117]). Rastogi *et al*. [117] likely have gotten inspired by Gang *et al*. [118], who suggested a similar possibility for CC3H, which has however also not been characterized as of yet. It would be of great interest to resolve this issue by characterizing (C)C4H’s ability to catalyse this reaction.

A potential second route involves the conversion of cinnamate into cinnamoyl-CoA, orchestrated by the ATP-dependent 4-hydroxycinnamoyl-CoA synthetase/ligase (4CL) (figure 2*a*), alongside potentially other acyl-CoA synthetases (ACS/ACoS), belonging to the large acyl-activating enzyme (AAE) [119]. While cinnamate is a poor substrate for plant 4CLs, 4CLs that use cinnamate and *p*-coumarate at equal catalytic rates occur in bacteria [120]—the extent to which this reaction occurs across streptophyte diversity remains to be investigated (dotted line in figure 2*a*). In the streptophyte algal progenitor of land plants, a clear orthologue of 4CL/ACoS was present [75]. Whether this enzyme worked as a *bona fide* 4CL cannot be inferred at present as functional characterizations of 4CL/ACoS co-orthologues found in streptophyte algae are lacking. Homologues related to 4CL are found across the green lineage [19]. *Arabidopsis* has an expanded 4CL family (four canonical (‘4CL’) and nine additional 4CL-like (‘4CLL’) members)—yet such lineage-specific radiations occurred several times independently among the 4CL/4CLL homologues [19]. Given the large repertoire of 4CL and 4CLL homologues—present even among some algae—the investigation of their repertoire in the LCA of land plants and algae becomes crucial. Overall, phylogenetic data support the presence of at least two 4CL/ACOS5-like genes at the base of Streptophyta [19]. Yet this step does not offer an alternative route if C4H is not capable of catalysing the conversion of cinnamoyl-CoA generated by the 4CL-dependent route to *p*-coumaroyl-CoA. Whether this reaction is catalysed by C4H or a convergently recruited CYP450 enzyme—which is an enormous family of enzymes known to be highly versatile [113,121]—remains to be investigated.

### (c) Lignin branch

Key for the structure of vascular plants is the large lignin polymers, whose monomeric building blocks derive from the phenylpropanoid pathway. An important group of enzymes in lignin-related processes, as well as in the synthesis of compounds with antioxidant and antimicrobial properties, is an enzyme belonging to the BEAT, AHCT, HCBT, DAT enzyme-acyltransferase family (BAHD acyltransferase family). Within the routes to lignin, the BAHD acyltransferase hydroxycinnamoyl-CoA:shikimate/quinate hydroxycinnamoyl transferase (HCT) catalyses the synthesis of shikimate esters by using *p*-coumaroyl-CoA as acyl donor [122]. Recent findings highlight the functional conservation of HCT in bryophytes and seed plants [123]. Already the LCA of land plants had an expanded repertoire of BAHD acyltransferase family members, including HCT [19,123]. These phylogenetic data further revealed streptophyte algal homologues, which, however, appear divergent from land plant HCT. This step catalysed by HCT is the second step in the Shikimate Ester Loop (SEL), introduced by Adams *et al*. [124]. It describes the four reactions necessary to convert *p*-coumaric acid to caffeic acid. Namely, the conjugation of CoA to *p*-coumaric acid by 4CL, the synthesis of *p*-coumaroyl-shikimate by HCT, conversion to caffeoyl-shikimate by *p*-coumaroyl shikimate 3′-hydroxylase (C3′H or CYP98), and finally the cleavage of shikimate by caffeoyl-5-*O*-shikimate esterase (CSE). The difference between the educt and product of the SEL is the *meta*-hydroxylation of the aromatic ring. This loop is hypothesized to serve a regulatory role, as shikimate is a necessary conjugate for these reactions to be catalysed, without being consumed itself. Shikimate, eponymous of the shikimate pathway (figure 1), is a precursor of phenylalanine, necessary for both the phenylpropanoid pathway as well as protein biosynthesis. The plastidially produced compound is shuttled to the cytoplasm, where its concentration determines the fate of phenylalanine. If photosynthesis occurs slowly or not at all (i.e. at night), the pool of cytosolic shikimate is small. Due to HCT’s low affinity for shikimate, very little will be conjugated to *p*-coumaroyl-CoA [125]. Hence, most shikimate and subsequent phenylalanine are used for protein biosynthesis in primary metabolism. When photosynthesis is occurring actively however, the cytosolic shikimate concentration increases, enabling the synthesis of *p*-coumaroyl shikimic acid by HCT [124,125]. C3′H (CYP98) acts on the product of HCT [126–128]. C3′H is part of the CYP450 family CYP98, which belongs to the extensive CYP clan CYP71 [113,121,128,129]. C3′H shows substrate promiscuity, facilitating hydroxylations of *p*-coumarate-derived compounds, vital for producing various phenylpropanoid-derived substances in land plants [130]. Within the phenylpropanoid pathway, it is a crucial enzyme and was detected in all land plants, while streptophyte algal homologues appear to code for rather divergent CYP450 enzymes [19,130]. Due to high substrate promiscuity in the CYP enzyme families and clans, alongside independent radiations of CYP450 enzymes in streptophyte algae, functional predictions are complicated. How lignin-like compounds of streptophyte algae are thus formed remains to be investigated.

The conversion of caffeoyl-5-O-shikimate to caffeic acid has been described as a crucial step in biosynthesis leading to guaiacyl (G)- and syringyl (S)-lignins, mediated by CSE [131]. In concert with 4CL/ACOS5, it can bypass the direct conversion of caffeoyl-shikimic acid to caffeoyl-CoA via HCT [131,132]. Yet some plants lack CSE orthologues: the model grasses *Brachypodium distachyon* and *Zea mays* have no CSE orthologue, and monocots in general probably lost them secondarily [133]. CSE in *Arabidopsis thaliana*, unlike other monoacylglycerol lipases (MAGLs), lacks hydrolytic activity on monoacylglycerols [134]—losing this activity might have accompanied or even caused its neofunctionalization as CSE. However, more functional work on phylodiverse CSE (and MAGL in general) homologues is needed. The MAGL family experienced early radiation in streptophytes, and the distribution of functionally characterized MAGLs from *Arabidopsis thaliana* implies ancient subfunctionalization events. Streptophyte algal MAGL homologues are associated with several MAGL clades, but their relation to the clade MAGL1/3 that includes CSE remains poorly resolved [19]. Additional, independent radiations in streptophyte algal lineages of MAGL sequences appear to have occurred, suggesting algal-specific subfunctionalization events [19].

In seed plants, ferulate-5-hydroxylase (F5H) and caffeate-O-methyltransferase (COMT) come into play in the pathway from *p*-coumaroyl-CoA to S-lignin, with COMT methylating caffeic acid or 5-hydroxyferulic acid produced by F5H [135,136]. The lycophyte *Selaginella moellendorffii* has convergently evolved F5H and COMT functions in CYP450 Clan 71 and COMT methyltransferases [137,138]. Genomic analyses of the lycophyte *Isoetes taiwanensis* show a lack of angiosperm F5H and COMT sequences but possess the lycophyte versions, suggesting that this change has occurred in the LCA of *I. taiwanensis* and *S. moellendorffii* [139]. Whether more and how often F5H and COMT function has evolved from phylogenetically distinct CYP450 enzymes and methyltransferases has not been studied. Caffeoyl-CoA O-methyltransferase (CCoAOMT) and its homologues catalyse the methylation of caffeoyl-CoA, leading to feruloyl-CoA [140,141]. The CCoAOMT-like protein family’s origin likely traces back to early Phragmoplastophyta, and exploring putative CCoAOMT-like enzymes in Coleochaetophyceae, which have been shown to produce lignin-like compounds [77,78] and Zygnematophyceae, which are the closest algal relatives of land plants [14,142], could unveil a synapomorphy with physiological relevance within the evolution of plant phenylpropanoid metabolism.

Phenylpropanoids undergo NADPH-dependent reduction of the acyl-group by cinnamoyl-CoA reductase (CCR); related NADPH-dependent reductases include dihydroflavonol reductases (DFRs) and DFR-likes (DFL) [143]. While streptophyte algae contain diversified CCR-like homologues, a putative assignment of them into CCR, CCRL, DFR and DFRL based on phylogenetic data alone is only possible for land plants [19]. Within land plants, a remarkable radiation of CCRs has occurred [19]. Before dedicated lignin biosynthesis commences, the cinnamyl alcohol dehydrogenase (CAD)-catalysed conversion of phenylpropanoid-derived aldehydes into alcohols. Based on flowering plants, CAD has been categorized into five groups [144]. The whole CAD family has undergone massive radiation, yielding large species- and lineage-specific clades. Also in chlorophytes and streptophytes are several CAD homologues and the LCA of embryophytes and algae might have had an expanded repertoire of CAD homologues—yet this is phylogenetically not well-resolved [19].

The phenylpropanoid pathway is a major carbon sink in land plants; it is plausible that its production of metabolites with diverse functions—that often come to bear only when accumulated to a high abundance such as structural polymers—only emerged during the course of evolution because of carbon being funnelled into these specialized metabolic routes [76]. This funnelling yielded a bouquet of compounds (and metabolic noise) on which evolution could act, fostering further diversification [76]. Non-vascular plants are rich in UV protectants whereas, due to forming the vascular tissues of our towering trees, lignin can arguably be considered one of the most abundant biomolecules on planet Earth [145]. However, the role of such polymers likely predate the origin of land plants. In land plants, a structurally and biochemically complex amalgamate of fatty acids, aromatics and polyketides exists that is called sporopollenin; the presence of hydroxylated polyketides produced by homologues of anther-specific chalcone synthase-like (ASCLs) has been proposed as the defining feature of the sporopollenin found in land plants [146]. This sporopollenin not only coats pollen but also the spores of non-flowering plants. Indeed, algae are known to produce a compound that is akin to sporopollenin called algaenan. Resilient coating with algaenan has been found to surround the reproductive structures of diverse streptophyte algae, including members of the Charophyceae [80,147], Coloeochaetophyceae [78] and Zygnematophyceae [148]. The latter has received detailed recent investigations [149–152], as scholarly reviews in this very special issue [152]. While the evolutionary relationship of algaenan and sporopollenin is in need of further investigation [146], it is conceivable that all of these resilience-conferring biopolymers share a common evolutionary origin [153].

### (d) Phenolic chemodiversity

CYP98 family members are not only involved in lignin biosynthesis as described above for C3′H, but other members of this CYP450 subfamily in angiosperms catalyse reactions with a diverse array of 4-coumaroyl esters or amides as substrates [130]. The subsequently formed hydroxycinnamic conjugates may be involved in plant defence against pests and pathogens, as they have been characterized as antiviral, antibacterial, anti-herbivory, anti-inflammatory and antioxidant [154]. Depending on the isoform of the CYP98, some are linked to lignin biosynthesis, pollen wall and -coat biosynthesis (feruloyl- and sinapoyl-conjugated phenolamides [153,155–157]) and soluble phenolics acting as UV protectants (chlorogenic acid [158]). Shikimate esters are proposed to be evolutionarily conserved intermediates involved in phenolic ring functionalization (and in angiosperms, this step is crucial) [123]. However, they are very transient intermediates which do not accumulate in the plant.

So far, we have given some account of what is currently known about the evolution of the enzymes involved in the production of phenolics. Remarkable is the overall modularity within the phenylpropanoid pathway. Multiple—if not most—enzymes exhibit a degree of promiscuity, always catalysing the same type of reaction on different substrates (figure 2*b*). For example, 4CL uses cinnamic, *p*-coumaric, caffeic, ferulic, 5-hydroxyferulic and sinapic acids and converts them to their respective CoA-thioester (figure 2*b*). This modularity has, already within land plants, led to a diversity of routes that lead to the same classes of metabolic products. As such, this modularity may well be the reason for the diversification of phenolic compounds within plants. Metabolic data on streptophyte algae are still scarce—and due to the lack thereof, the available databases are land plant-centric. As of yet, it thus is unknown how phenolic biosynthesis has diversified in streptophyte algae and how different the committed enzymes at the different modular steps are—that is, how much convergent evolution might have occurred.

Another well-researched and evolutionary exiting route deriving from the phenylpropanoid pathway is that of flavonoids. The evolution of the genes involved in flavonoid biosynthesis is nicely reviewed in another review included in this special issue, by Agorio *et al*. [159]. We will thus not further go into detail regarding flavonoid biosynthesis in our review but rather would like to end this review by discussing an evolutionary scenario that might have shaped the phenolic metabolome of land plants and algae in the terrestrial habitat.

## 5. The pull

Metabolites produced by the phenylpropanoid pathway serve an obvious advantage in surviving the harsh terrestrial habitat, not least due to their relevance to stress [27] but also for structural purposes [132]. Hence, even in plants thriving on land without the elaborate phenylpropanoid pathway known in vascular plants, phenolic compounds are present to respond to environmental factors. We can assume that they are under selection. Let us unpack this a bit. What gets selected? The phenotype. The phenotype in our treatise is an effective cocktail of phenolics that can ward off adversaries—both of biotic as well as abiotic nature. Both occur simultaneously, and it is the mixture of compounds that shapes the concerted action towards various cues—with overlapping and distinct roles. Similar to genes, there can thus be redundancy in the compounds. When the selective pressures come to bear on this cocktail, the answer to challenges is an adequate mix.

**Figure 3.**
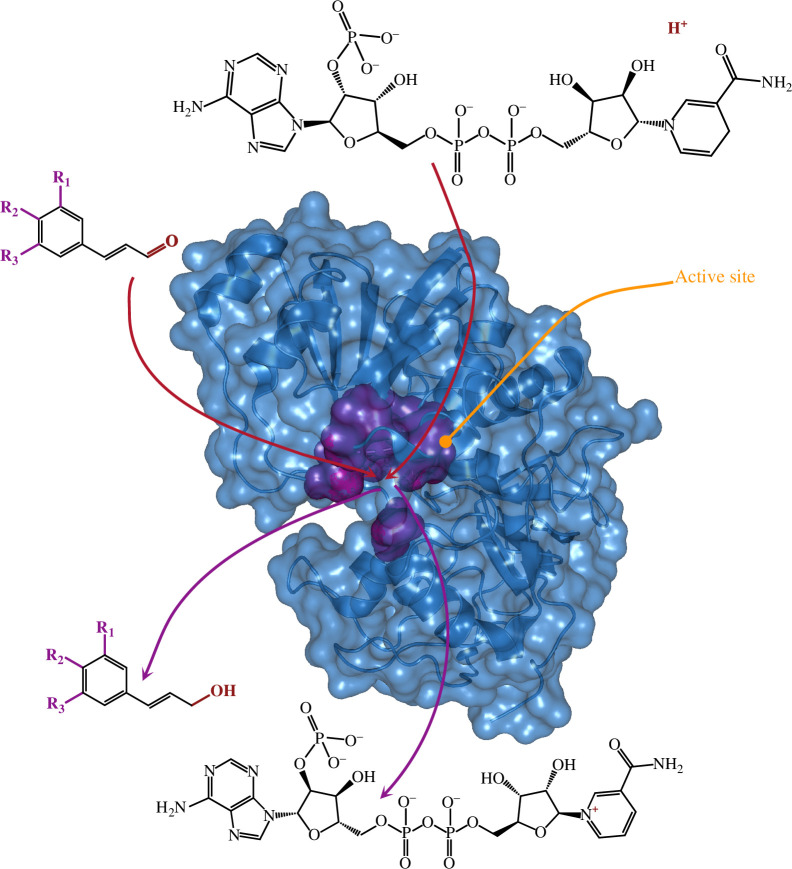
Promiscuity and radiation. Enzyme model based on CAD [160]. The backbone is indicated in dark blue, surface in lighter blue. The surface of active site residues is indicated in purple. Arrows indicate active site (orange), substrate influx (red), and product efflux (purple). Cinnamaldehyde derivative substrates can be converted to cinnamyl alcohol derivative products irrespective of their specific substituents, either alcohol or methoxy groups. Necessary commonality is indicated in bold red (aldehyde/alcohol group), and possible versatility is indicated in bold purple (R_1-3_). This promiscuity towards substrates of enzymes involved in the phenylpropanoid pathway and its branches may have led to (or even driven) the evolution of this pathway.

This phenotype, containing an effective cocktail of phenolics to ward off adversaries, both of biotic and abiotic nature, may have partly evolved neutrally or due to drift, and later experienced selective pressure, where this adequate mix was advantageous [76]. As there are thousands of phenolic compounds that derive from the shikimate pathway in plants [43], some compounds are possibly redundant. This redundancy and functional diversification of the compounds lead to metabolic plasticity. This can be achieved by catalytically promiscuous enzymes as part of the evolving pathway, associated with neo- and subfunctionalization, as described for the shikimate pathway, serving as an example of ancestral-like promiscuity (figure 3) [161].

In addition to possible enzymes more distantly related to the enzymes known to catalyse specific reactions in vascular plants, alternative biosynthetic routes may have evolved, yielding the same product [137]. As illustrated, in the case of the zygnematophyceaen alga *Penium margaritaceum*, despite a patchy phenylpropanoid pathway, a homologue to an enzyme converting a downstream product is present (CHS, converting coumaroyl-CoA [22]). This convergent evolution towards phenolic compounds most likely still uses the same compounds, derivatives of the shikimate pathway (figure 1). This raises an important aspect: the question of how both convergent and parallel evolution shapes the production of plant phenolics. Imposed by external cues that drive the selection for specific properties, convergence in enzyme functions can be easily envisioned. However, given the promiscuity concomitant to the early emergence of certain enzyme families, parallel evolution can be conceived that makes use of the same evolutionary substrate yielding, in the end, the same routes. This might even extend to the compounds themselves. Versatility in enzyme evolution yields an advantageous end product, but how the organism produces it is of secondary priority – there is not only one enzyme, one gene sequence responsible and able to arrive there. These independently emerged biosyntheses can take the same route (parallel), different routes that end up with the same product (convergence in enzyme function), and different routes that yield a different molecule that, however, fulfils the same function (convergence in metabolite function).

Following the availability of phenolic compounds in the LCA of land plants, it is conceivable that the functionality of these compounds, advantageous to stress resistance of both biotic and abiotic nature, was later co-opted for a different function altogether: as UV-protective monomers to the formation of structurally supportive and water-conducting polymers—lignin. The first steps would have been the accumulation and transport of phenolic compounds to the cell wall, where they could polymerize. Hence, these polyphenols may have been transported to the cell wall to act as UV-protective shields, where they eventually polymerized. It has been speculated that these polyphenolic compounds may resemble lignin to some extent [162]. This may have been advantageous for more reasons than just UV protection, leading to structural support which was beneficial and aided in competition for light. The water-conducting ability of lignin evolved in the LCA of vascular plants, no longer being the prime factor for surviving on land, as bryophytes prove.

## Data Availability

This article has no additional data.
